# Intraoperative radiotherapy with low energy x-rays for primary and recurrent soft-tissue sarcomas

**DOI:** 10.1186/s13014-020-01559-7

**Published:** 2020-05-14

**Authors:** Gustavo R. Sarria, Vera Petrova, Frederik Wenz, Yasser Abo-Madyan, Elena Sperk, Frank A. Giordano

**Affiliations:** 1 Department of Radiation Oncology, University Medical Center Mannheim, Medical Faculty Mannheim, Heidelberg University, Theodor-Kutzer-Ufer 1-3, 68167 Mannheim, Germany; 2 University Medical Center Freiburg, Medical Faculty Freiburg, Freiburg University, Freiburg, Germany; 3 Department of Radiation Oncology, University Hospital Bonn, University of Bonn, Venusberg Campus 1, 53127 Bonn, Germany

**Keywords:** Soft-tissue sarcoma, Electronic intraoperative radiotherapy, IORT, Kilovoltage, Low energy x-rays

## Abstract

**Background:**

Soft tissue sarcomas (STS) treatment remains a therapeutic challenge. Intraoperative radiotherapy (IORT) resembles a safe and efficient for STS treatment. The first data on electronic-IORT (eIORT) using low-energy photons is herein presented.

**Methods:**

Thirty-one patients with newly and recurrent STS were retrospectively assessed. EIORT was applied with low-energy photons during surgery. The dose was either prescribed to the applicator surface (spherical applicators) or 5 mm depth (flat applicators). Overall progression-free survival (O-PFS), local progression-free survival (L-PFS), overall survival (OS) and adverse events were evaluated.

**Results:**

Median follow-up was 4.88 (1.0–8.95) years. Twenty-five patients (80.6%) had recurrent STS with prior treatment. The resection status was R1 in 25.8% and R2 in 6.5%. The distribution was 51.7% for extremities, 35.5% for abdomen and pelvis, 9.7% for thorax and 3.2% for head and neck tumors. The median O-PFS was 11.0 months, with 42.6% 5-year estimated O-PFS. The only local recurrence in the primary setting occurred after 22 months. Median L-PFS in recurrent STS was 12.5 months, with 65.5% 5-year estimated L-PFS. The 5-year OS estimated rate was 94.7% (3 events after 7 years). No G3 toxicity related to eIORT was observed. Two patients exhibited G2 acute neuropathic pain. Late neuropathic pain was seen in 6 patients being 3 graded as G1 and 3 as G2. No wound-related toxicity was found.

**Conclusion:**

Electronic IORT with low-energy photons is a safe treatment option for STS, yielding similar outcomes as historical series reporting IORT with electrons or HDR brachytherapy.

## Introduction

With an approximate overall incidence of 6/100000 [1] and less than 1% of the all-cancers global prevalence, sarcoma is a rare tumor entity [2]. However, it accounts for 13.040 new patients per year in the US [3] and the overall lethality of this tumor is still high [4].

Tumor recurrence usually occurs within the first 18 months of treatment and depends on many variables such as primary location, histology, stage or resection status [5–7]. The cornerstone for sarcoma management remains to be the upfront surgical approach and the addition of radiotherapy in cases where needed [8, 9], according to a variety of features listed in different international guidelines [10], which enhances the therapeutic ratio compared to a single-intervention approach, as randomized trials have previously reported with an overall local control rate increase of 20–25% [11, 12]. In consequence, treatment strategies may vary depending on these factors [13]. Historical data shows that approximately 50% of patients with primary retroperitoneal tumors do not achieve a complete resection due to anatomic challenges and even in case tumors were completely resected, patients still exhibit a 50% risk for recurrence [13]. Thus, the nature of this tumor also represents a challenge for the radiation oncologist, as the best response outcomes after radiotherapy are seen after complete resection [14, 15]. Regarding extremity sarcomas, the greatest benefit of this treatment might be related to limb-sparing surgery, as the increased local control yields in lower recurrence rates, therefore fewer salvage amputations would be needed [16].

Amongst all therapeutic options, intraoperative radiotherapy (IORT) has been used for additional tumor bed treatment for more than 50 years as it results in improved outcomes in terms of local control [17, 18]. However, IORT was either applied using forward-directed electron beams or using high-dose rate (HDR) brachytherapy with flexible applicators (“flabs”) that were placed on the tumor bed and then after-loaded with ^192^Ir or ^60^Co sources [19, 20]. A further, more recent intraoperative treatment option is the use of a portable linear accelerator delivering low-energy (50 kV) photons [21]. This system is FDA-approved for treatment of all tumors within the human body has it proven safe and efficient in a variety of phase I-III trials [22–25]. We treated a total of 31 patients with primary and recurrent soft-tissue sarcoma with this novel modality and here for the first time report the results of a retrospective analysis of their outcome.

## Methods

### Patient selection and procedures

Patient charts of 31 patients with primary and recurrent soft-tissue sarcoma STS treated at our institution between December 2008 and June 2017 were retrospectively analyzed. All patients underwent surgery after MRI-based diagnosis establishment and preoperative feasibility assessment. During resection, patients were treated (Intrabeam, Carl Zeiss Meditec AG, Oberkochen, Germany) with either a forward-beaming flat applicator (providing dose uniformity [“flatness”] perpendicular to the beam direction greatest at the prescription depth of 5 mm) or a ball-shaped applicator providing a spherical isotropic dose distribution [21, 26], for which the resection borders were approached with a purse-string-like suture. Depending on region, risk structures and previous EBRT irradiation, electronic IORT (eIORT) was delivered with doses between 5 and 20 Gy prescribed to 5 mm (flat applicator) or at the applicator surface (spherical applicators). Doses were chosen after these considerations and clinician’s discretion. The applicator was selected in-situ according to the surgical bed morphology, assuring an adequate coverage of the intended area to treat. The region that was judged clinically to be the area with highest likelihood of residual tumor or R2 situation under direct observation was defined as target volume, as discussed between the surgeon and the radiation oncologist and based on the limitations for further resection.

This study was approved by local Ethics committee, according to institutional protocols. All patients gave informed consent prior to treatment application. All ethics standards were applied according to the Declaration of Helsinki.

### Outcome assessments

Factors of interest were age, gender, location, histology, previous and subsequent therapies as well as response determined in regular follow-ups including imaging. Staging was performed through MRI and thorax-CT. All patients undergoing IORT were scheduled for 1st-, 3rd-, 6th -, 12th -, 18th,- 24th-month and afterwards yearly post-treatment follow-ups, with 6-month interval MRI and thorax-CT during the first 2 years and annual thereafter. Progression-free survival (PFS) and overall survival (OS) were defined as time from eIORT until tumor recurrence at any site or death by any cause. Local PFS (L-PFS) was defined as time from eIORT until local tumor recurrence or death event. The entire tumor bed was considered for L-PFS purposes. Toxicity was assessed according to the Common Terminology Criteria for Adverse Events (CTCAE, v. 5.0). The cut-off between acute and late toxicities was defined to be 3 months after eIORT. All statistical analyses were carried out using SPSS (Version 24.0., IBM, Armonk, NY), and significance was assess as per the t-test.

## Results

### Patient collective

We analyzed charts of 15 female and 16 male patients (Table 1). The median follow-up was 4.88 years (range: 1.0–8.95 years). The median age was 48 years (15–77 years). The mean tumor size was 7.1 cm (1.8–22). Of all 31 patients, 16 had extremity sarcoma (51.7%), 14 had a diagnosis of pelvic and abdominal tumors (35.5%), 3 had thoracic sarcoma (9.7%) and 1 patient had a head and neck primary sarcoma (3.2%). Twenty-five patients (80.6%) had recurrent disease and were previously treated (surgery and/or external beam radiotherapy [EBRT]). Four patients with previously un-irradiated recurrences received neoadjuvant EBRT. The post-recurrence surgical margin status was found to be R0 in 18 patients (58.1%), R1 in 8 patients (25.8%), R2 in 2 patients (6.5%), and not clear in 3 patients (9.7%).

**Table 1:** Patient characteristics

Characteristic	N	%
**Age**		
Median Age (Range) [years]	48 [15–77]	
**Gender**		
Male	16	51.6
Female	15	48.4
**Disease Status**		
Primary	6	19.4
Recurrent	25	80.6
**Tumor Location**		
Upper extremity	3	9.7
Lower extremity	13	42
Abdomen/pelvis	11	35.5
Thorax	3	9.7
Head and neck	1	3.2
**Histology**		
Liposarcoma	9	29.0
Leiomyosarcoma	3	9.7
Myxofibrosarcoma	3	9.7
Neurofibrosarcoma	4	12.9
Others	12	38.7
**Grading**		
G1	6	19.4
G2	8	25.8
G3	8	25.8
Unknown	9	29.0
**Tumor Size**		
Mean/Range [cm]	7.1 [1.8–22.0]	
**Prior RT**		
Yes	13	41.9
No	18	58.1
**Resection Status**		
R0	18	58.1
R1	8	25.8
R2	2	6.5
Unknown	3	9.7
**IORT**		
Spherical applicator	28	90.3
Flat applicator	3	9.7
Dose (Range) [Gy]	5–20	
**EBRT after IORT**		
Yes	15	48.4
No	16	51.6
**Systemic therapy after IORT**		
Yes	9	29.0
No	22	71.0

### Efficacy outcomes

**Fig. 1:**
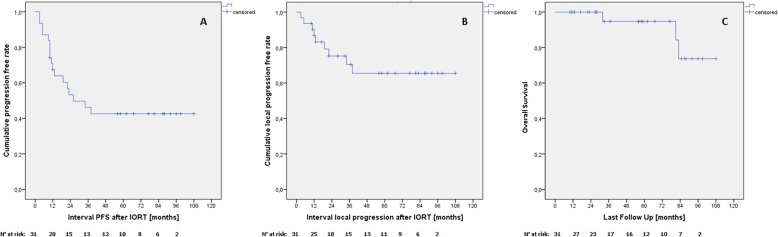
Outcomes per the entire cohort. **a** Overall progression-free survival. **b** Local progression-free survival. **c** Overall survival

**Fig. 2:**
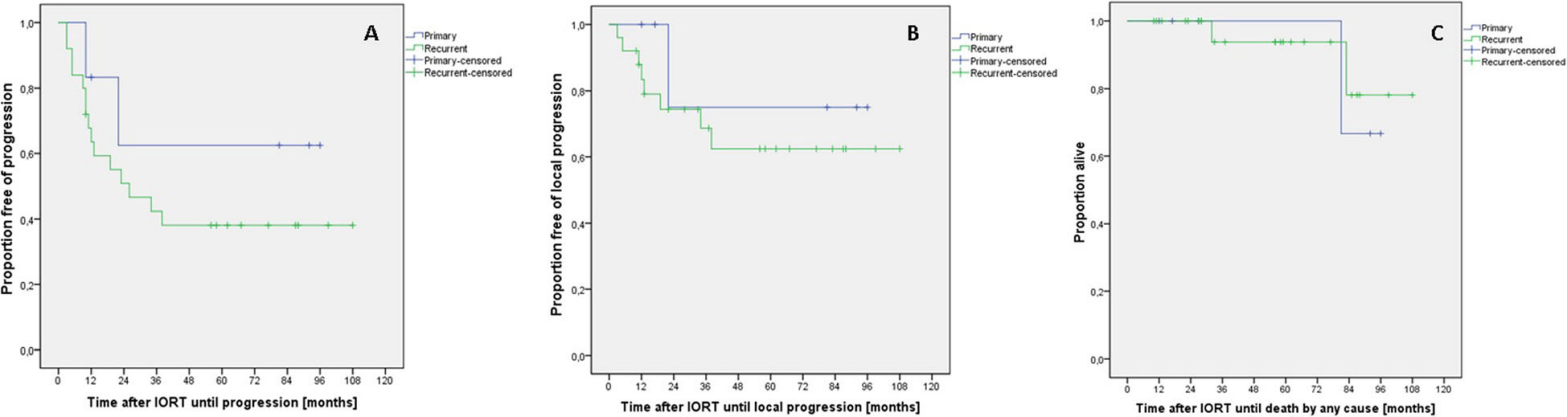
Outcomes according to primary/recurrent status. **a** Overall progression-free survival. **b** Local progression-free survival. **c** Overall survival

The median O-PFS was 11.0 months for both primary and recurrent tumors (range: 10–12 months for primary and 3–38 months for recurrent tumors). The 5-year estimated O-PFS was 42.6% Fig. 1a (62.5 and 38.1% for primary and recurrent [*p* = 0.316], respectively, Fig. 2a). Distant metastasis events were recorded in 7 recurrent STS patients and 1 primary STS patient after 10 months. Only one local recurrence occurred in the group of patients with primary STS after 22 months and 8 in the recurrent group. The median L-PFS in the group with recurrent STS was 12.5 months (3–38 months) and the estimated 5-year L-PFS was 65.5% (Fig. 1b and Fig. 2b). The 5-year OS estimated rate was 94.7% Fig. 1c (1 event for primary and 2 events for recurrent [*p* = 0.725], c). There was no statistically significant difference amongst anatomical location in terms of O-PFS (*p* = 0.685), L-PFS (0.998) or OS (*p* = 0.444).

### Toxicity profile

The toxicity profile shows that no G3 toxicity occurred related to eIORT. Two patients (6.45%) exhibited acute G2 neuropathic pain. Late neuropathic pain was seen in 6 patients (12.9%), with 3 being graded as G1 and 3 as G2 in severity, without any functional impairment and complete resolution after medical treatment. No wound-related toxicity was found **(**Table 2**)**.

**Table 2:** Toxicity

Toxicity (CTC AE Grade)	N (events)	%
**Acute toxicity**		
Grade 0	22	71.0
Grade 1	2	6.5
Grade 2	0	0
Grade 3	0	0
Grade 4	0	0
Not reported	7	22.6
**Late toxicity**		
Grade 0	15	48.4
Grade 1	3	9.7
Grade 2	3	9.7
Grade 3	0	0
Grade 4	0	0
Not reported	10	32.3

## Discussion

Although the improvement in operative and adjuvant treatment techniques during the past decades, sarcomas are still associated with an increased risk of mortality amongst all cancers [27, 6]. Under our hypothesis, for which IORT provides improved metastasis-free and overall survival, besides the already proven local control benefit, we sought to intensify local therapy by the addition of eIORT, as surgery-alone or perioperative EBRT-alone historical data have shown mixed data regarding these outcomes [28, 11, 12, 29].

Despite the fact that the vast majority of our patients (> 80%) presented with recurrent sarcoma, the local control rates appear specifically encouraging in comparison with previous data from other limb-sparing approaches [30, 7, 31]. Although there is a high variability of L-PFS, D-PFS and OS rates due to multiple factors (such as location, resection margins, grade, etc.), the 5-year OS rate seen in our collective (83.3–92%) compares favorably with previous reports on IORT for recurrent sarcoma:

*Call* et al. reported on outcomes of 61 patients with upper-extremity sarcoma treated with surgery plus IORT (electrons or HDR), with the majority of patients being diagnosed with primary tumors (53 patients, 87%). The 5 year local control for previously untreated and recurrent tumors was 94 and 67%. Distant control and overall survival (OS) rates were 82 and 70% for newly diagnosed and 74 and 63% for recurrent tumors. Interestingly, there was no significant difference regarding the resection status, R0 or R1, for all 3 endpoints evaluated, indicating efficacy of the approach. Four patients experienced severe adverse events that were judged to be related to IORT (2 wound complications, 1 severe neuropathy and 1 vascular necrosis of the humeral head) [32].

*Haddock* et al. reported the outcomes of 91 patients with primary (*n* = 74) or recurrent (*n* = 17) limb and girdle sarcomas, who underwent perioperative EBRT and electron-based IORT (IOERT). After a median follow-up of 2.5 years, the L-PFS was 92%. Twenty-three patients developed distant progression, mostly pulmonary. The OS at 3 years was 76%. In this analysis, a statistically significant difference was found between resection status and local control [33].

Recently *Roeder* et al. analyzed 183 patients with primary limb sarcoma (78% primaries, 22% recurrent) treated with IOERT at an average dose of 15 Gy [34]. After a median follow-up of 64 months, the L-PFS rates at 5 and 10 years were 86 and 84%, whereas the resection status also influenced this in great fashion (5-year L-PFS rates were 92% vs 75% for R0 and R+). Similarly, primary and recurrent tumors responded differently with 5-year L–PFS rates of 90 and 74% [34].

*Calvo* et al. described a potential benefit for high-risk features in patients who underwent electron-IORT with incomplete or close resection margins in comparison to historical reports on post-surgery EBRT-only patients. The L-PFS and OS rates after 5 years were 82 and 72% respectively, which could be compared to patients with more favorable features who showed L-PFS and OS rates of 72–96% and 71–87% respectively [35].

Another report from *Roeder* et al. addressed the outcomes of 27 patients treated in primary or recurrent scenarios for retroperitoneal sarcomas. Most of the patients had neoadjuvant EBRT (median dose 50 Gy). The margins status was complete (R0) in 22% of the cohort and margin-positive (R1) in 74% of the patients. Out of this group 85% received IORT to a median dose of 12 Gy. With a median follow-up of 33 months, the L-PFS was 72%, O-PFS 63% and OS 74% at 5 years-estimated analysis [36].

All these results show good outcomes in terms of local control despite the increased rate of positive resection margins (R1-R2) and recurrent nature of the malignancies, which are similar to the ones registered in this study. This reinforces the importance of IORT in high-risk features scenario.

Similarly to the previously mentioned publications, the dose range for IORT should be fixed in the range of 10 to 20 Gy, according to the recently published American Brachytherapy Society recommendations [37]. The selected doses for our cohort are supported by multiple factors, such as previous irradiation, organs-at-risk exposure and the relative biological effectiveness (RBE) of kilovoltage therapies. An adopted RBE factor of 1.3 has been standardized in our institution, based on published evidence regarding this topic [38]. Additional considerations must be taken into account depending on the selected applicator. In cases were a flat applicator was employed, the normalizations was done at 5 mm [21]. For the spherical applicators, a dose prescription is superficially determined; however, a dose distribution of approximately 30% is reached at 1 cm distance [38]. All this factors should be kept present when prescribing eIORT management.

As for OS only a few trials have addressed this evaluation. A recently published systematic review by *Albertsmeier* et al. has described a significant OS benefit in patients with retroperitoneal sarcomas who underwent prior EBRT to surgery, analyzing 554 patients with an OR of 0.37 (*p* < 0.00001). They describe as well that this benefit would be potentially greater in patients with positive resection margins. Despite these findings, there was no benefit demonstrated out of 2428 patients who had primary limb sarcoma diagnosis, although local control shows better outcomes [39, 40]. Our study shows really encouraging OS outcomes for a cohort conformed by mostly recurrent patients. These results might be supported by many factors, such as the improvement of healthcare for metastatic patients, localized radical radiotherapy and systemic treatments, yielding an overall improvement of survival rates [41]. An additional factor not to be disregarded is the idiosyncrasy of these tumors, as they develop in more extended time periods, compared to other malignancies.

For metastatic-scenario patients, current recommendations point to radical primary treatment and aggressive targeted treatment depending on the clinical status [42]. A new therapeutic window appears, as some papers have described a potential benefit of triggering the bystander and abscopal effect by high-dose delivery to primary or metastatic tumor areas. Intraoperative radiation therapy emerges as a good alternative due to its conformal nature and good tolerability with less expected secondary effects as SBRT deliver. Although more data is warranted, initial reports are encouraging due to their favorable outcomes in survival benefit [43–45].

Another, although less commonly used method of IORT is HDR brachytherapy with multi-catheter placement or flab applicators, which allow shaping the target area as the anatomy presents, at the same time prescribing precisely the extension of margins desired. Some of the longest follow-up date has been published based on HDR and electron-IORT experiences, which show solid data supporting this approach [46, 47]. However, the logistics are challenging as surgical theaters must be adequately shielded due to radiation protection requirements [48].

Low-energy photons have reappeared in scene for the past few years, as different trials have shown their versatility for eIORT approach in many different scenarios as primary breast, brain, skin, metastatic, amongst others described [22, 23, 49]. Due to its nature, the portable linear accelerator can be used without major prerequisites into mostly any surgical theater (e.g. that is cleared for C-arm fluoroscopy), as the 50-kV nominal energy delivered plus local shielding is more than sufficient for accomplishing radioprotection regulations [26]. The availability of different applicators allows shaping to most of the surgical beds as needed. In our series, the most commonly used applicator was the spherical applicator, as it allowed covering the entire cavity in 360° by approaching the surrounding tissues together.

The sharp dose fall-off of low-energy photons is adequate for avoiding most risk structures and protecting the surrounding normal tissue [50] to keep the incidence of toxicity low [32].

The toxicity profile seen in our analysis was deemed acceptable, as no severe complications pre or post-operative were seen and only G2 toxicity was found during follow-up, considering that a large proportion (*n* = 14) of this cohort debuted with intracavitary primaries, which is also reported in similar fashion by other groups. This might be explained by the limited tissue irradiated with IORT, as larger EBRT volumes could be avoided, which is of specific interest in the recurrent setting.

In our series, 25 patients were previously treated, whereas 10 of those remained without any evidence of local recurrence during follow-up, considering the recurrent nature of these tumors and accounting that over than 30% of this group had positive resection margins. No severe toxicity was developed during controls. It’s worth mentioning that, amongst the limitations of this approach, the coverage of an entire surgical bed might not be possible in many cases, therefore a thorough follow-up assessment of the selected area to treat must be considered. However, selecting the high-risk area to treat in a large resection cavity should not raise greater concern, due to the large experience and generated evidence through the years with this very same technique application [51].

## Conclusion

Taken together, with all limitations of a retrospective study and a heterogeneous cohort of patients, eIORT for limb and intracavitary STS seems to be of benefit regarding safety profile and L-PFS. Prospective evaluation is warranted to confirm these findings.

## Data Availability

Data not available.

## Abbreviations

STS: Soft-tissue sarcoma
IORT: Intraoperative radiotherapy
eIORT: Electronic intraoperative radiotherapy
O-PFS: Overall progression-free survival
L-PFS: Local progression-free survival
OS: Overall survival
HDR: High-dose rate brachytherapy
EBRT: External-beam radiotherapy
IOERT: Electron-based intraoperative radiotherapy
